# Adherence to the test, treat and track malaria policy among selected health facilities in Ghana: the clients’ perspective

**DOI:** 10.1186/s12936-025-05467-7

**Published:** 2025-10-14

**Authors:** Charles Kyei, Charlotte Tawiah, Francis Agbokey, Japhet Adomako Anim, Mathilda Tivura, Samuel Afari-Asiedu, Clifford Kwarteng Kyeremateng, Francis Mensah Kornu, Joshua Oppong, Wilfred Zuuri, Isaac Nyampong, Vitalis Atambila Agana, Prince Imoro Awimba, Annobah-Sarpei Nii Ankonu, Elizabeth Awine, Paul Welega, Stephaney Gyaase, Richard Joshua Tetteh, Peter Mayers, Samuel Bernard Ekow Harrison, Kwaku Poku Asante

**Affiliations:** 1 Kintampo Health Research Centre, Kintampo (KHRC), Research and Development Division (RDD), Ghana Health Service (GHS), Accra, Ghana; 2 Alliance for Reproductive Health Right (ARHR), Accra, Ghana; 3 Anglican Diocesan Development Relief Organization (ADRRO), Bolgatanga, Ghana; 4 Dodowa Health Research Centre (DHRC), RDD, GHS, Dodowa, Ghana; 5 Navrongo Health Research Centre (NHRC), RDD, GHS, Navrongo, Ghana; 6 Management Systems International (a Tetra Tech Company), Courthouse, Arlington, VA USA

**Keywords:** Adherence, T3 policy, Clients’ perspective, Malaria epidemiological zones, Ghana

## Abstract

**Background:**

Malaria continues to be a major disease burden affecting all ages. The WHO in 2012 introduced the test, treat and track (T3) policy for malaria management in endemic settings. All malaria suspected conditions are to be confirmed by test and treatment initiated with recommended artemisinin-based combination therapy (ACT) and treatment outcomes monitored over the course of the illness. This study evaluated the adherence of health facilities and client’s perspective of T3 policy in Ghana.

**Methods:**

This cross sectional study was conducted in November 2019 involving 30 health facilities conveniently selected from six districts in the 3 malaria epidemiological zones, and clients exit interviews were performed from each facility. Factors associated with the *test*, *treat* and *track* defined outcomes were assessed using chi square and multivariable logistic regression models at 5% level of significance and 95% confidence interval. Data were classified according to facility and clients’ perspectives.

**Results:**

Overall, 590 patients and 30 health facility managers were interviewed from 30 facilities in 6 districts across the three zones. CHPS compounds formed 18 (60.0%) of facilities assessed. Twenty-nine out of 30 health facilities had Rapid Diagnostic Test (RDT) kits and antimalarials. Health facilities in the southern zone of Ghana had 2.87 increased odds of adhering to the T3 policy compared to the middle zone [aOR = 2.87 (1.7, 4.8): p < 0.001]. Males were more likely not to return to the health facility for review or more likely to miss home visit [aOR = 0.6 (0.3, 0.9): p = 0.018].

**Conclusion:**

Testing and treating for malaria were high among health facilities in the three zones. However, tracking of patients was very low across the zones. Adherence from clients’ perspective was low especially for males. The study recommended among others that the National Malaria Elimination Programme (NMEP) should ensure periodic trainings of health facility staff especially those within the Northern and Middle zones of Ghana and strengthen monitoring and supervision of health facilities to enhance adherence to the T3 policy.

## Background

Malaria continues to be a major public health problem that affects people of all ages. An estimated 228 million cases of malaria were reported globally in 2018, with the majority of those cases (213 million, or 93%) occurring in the World Health Organization (WHO) African Region [1]. Also, the proportion of malaria among every five children sampled is 46.7 in Ghana [2]. As global health goals are continuously being embedded into country level healthcare policies, governments and relevant health stakeholders must collectively work towards bridging gaps within the management and control of malaria by focusing on patient-centred care. With the 2030 Sustainable Development Goals in mind, particularly SDG 3.3 which seeks to end epidemics of malaria globally, it is important that health care facilities effectively adopt strategies to combat malaria.

The WHO in 2012 introduced the test, treat and track (T3) policy to standardize the process of malaria management in endemic settings. This policy urges the universal testing of all suspected cases of malaria, regardless of age or endemicity, the treatment of positive test cases with anti-malarial medications of high quality, and the tracking of all patients who have been confirmed and treated for malaria [3]. Ghana adopted the T3 policy in 2013, mainly to ensure accurate diagnosis and treatment of malaria in Ghana and to reduce pressure on antimalarial medicines by ensuring that only confirmed cases are given treatment [4]. The WHO then recommended the prescription of artemisinin-based combination therapy (ACT) for those who test positive for malaria [5]. It is, therefore, important to note that prompt and accurate diagnosis and treatment of malaria are essential to reduce disease progression to severe disease and complications [6].

The T3 strategy which is one of the proposed WHO strategies to combat malaria anchors the key policy messages of the WHO’s recommendations on diagnostic testing, treatment and surveillance, i.e. that every suspected malaria case should be tested, every confirmed case should be treated with a quality-assured antimalarial medicine, and the disease should be tracked through a timely and accurate surveillance system. It requires a continuous evaluation of the effectiveness of strategies employed to combat the disease [7].

Seven years following the policy’s introduction, an evaluation of its effectiveness was required at various facility levels and in the neighborhoods where malaria is most prevalent. This will facilitate the development of target focused policies and advocacy that can ensure the eradication of malaria. It also seeks to add to existing literature that documents the best practices which adoption by health facilities would ensure that patients testing positive for malaria are treated and tracked to prevent relapse after accessing malaria services. The information gathered for the study will help stakeholders re-strategize and streamline their projects to contribute to government’s effort to achieve the national T3 policy. Policymakers will understand the factors that hinders the tracking system on the part of the healthcare provider and that of the clients as well.

This study therefore evaluated the adherence from the client’s perspective on the T3 policy in selected health facilities across the three malaria epidemiological zones in Ghana. Looking at the processes so far, it is essential that each component of the T3 policy pillars: *test*, *treat* and *track* is fully assessed to understand the main enablers and challenges to achieve optimum outcomes. The evidence from this study provides very vital information for policymakers to decide on the best supportive intervention to the T3 policy.

## Methods

### Study design

This was a cross sectional study where health facility managers were interviewed and clients exit interviews conducted with patients who had febrile conditions from health facilities in six districts across the three epidemiological zones in Ghana.

### Study setting

The study was conducted in six Districts/Municipalities; in the three malaria epidemiological zones, the northern savannah zone, the middle/transitional and forest zone, and the coastal savannah zone are Ghana’s three malaria epidemiologic zones [8]. The intensity of malaria transmission in the country is intermediate in the center transitional/forest zone, lowest in the coastal shrub zone, and highest in the northern savannah zone. The prevalence of *Plasmodium falciparum* infections and the frequency of mosquito bites, which differ throughout the three ecological zones, are linked to this variation in severity [9] (Table 1).

**Table 1:** Selected districts and epidemiological zones

No	District	Zone	Characteristics
**1**	Mpohor District	Coastal Savanna Zone (Southern Zone)	Rural
2	Nzema East Municipality	Coastal Savanna Zone (Southern Zone)	Peri-Urban
3	Kintampo North Municipality	Middle/transisitonal forest Zone (Middle Zone)	Urban
4	Kintampo South District	Middle/transisitonal forest Zone (Middle Zone)	Rural
5	Jirapa District	Northern Savanna Zone (Northern Zone)	Rural
6	West Mamprusi Municipalities	Northern Savanna Zone (Northern Zone	Rural

*Source* The table reports were developed during data collection

Mpohor District and Nzema East Municipal are both located in the Western North Region. The two districts are largely rural and have a total population of about 147,094, majority of which engage in fishing, agro-processing and mining [10]. The area is highly malaria endemic and has a doctor-patient ratio of 1:21,461. There are 2 district hospitals, 5 health centers, and 9 CHPS compounds.

The Kintampo North Municipality and Kintampo South District are located within the forest-savannah transitional ecological zone in the Bono East region of Ghana. The two districts together cover an area of 7162 km^2^, and largely rural with a resident population of approximately 228,631 who are predominantly engaged in subsistence farming [10]. Public health facilities in the 2 districts include 2 hospitals, 12 health centers/clinics, and 30 CHPS compounds; whilst the privately-owned health facilities include 4 clinics, 2 maternity homes, 4 pharmacies, and 86 Over the Counter Medicine Sellers (OTCMS) [11]. The area has a high perennial incidence of endemic malaria.

**Fig. 1:**
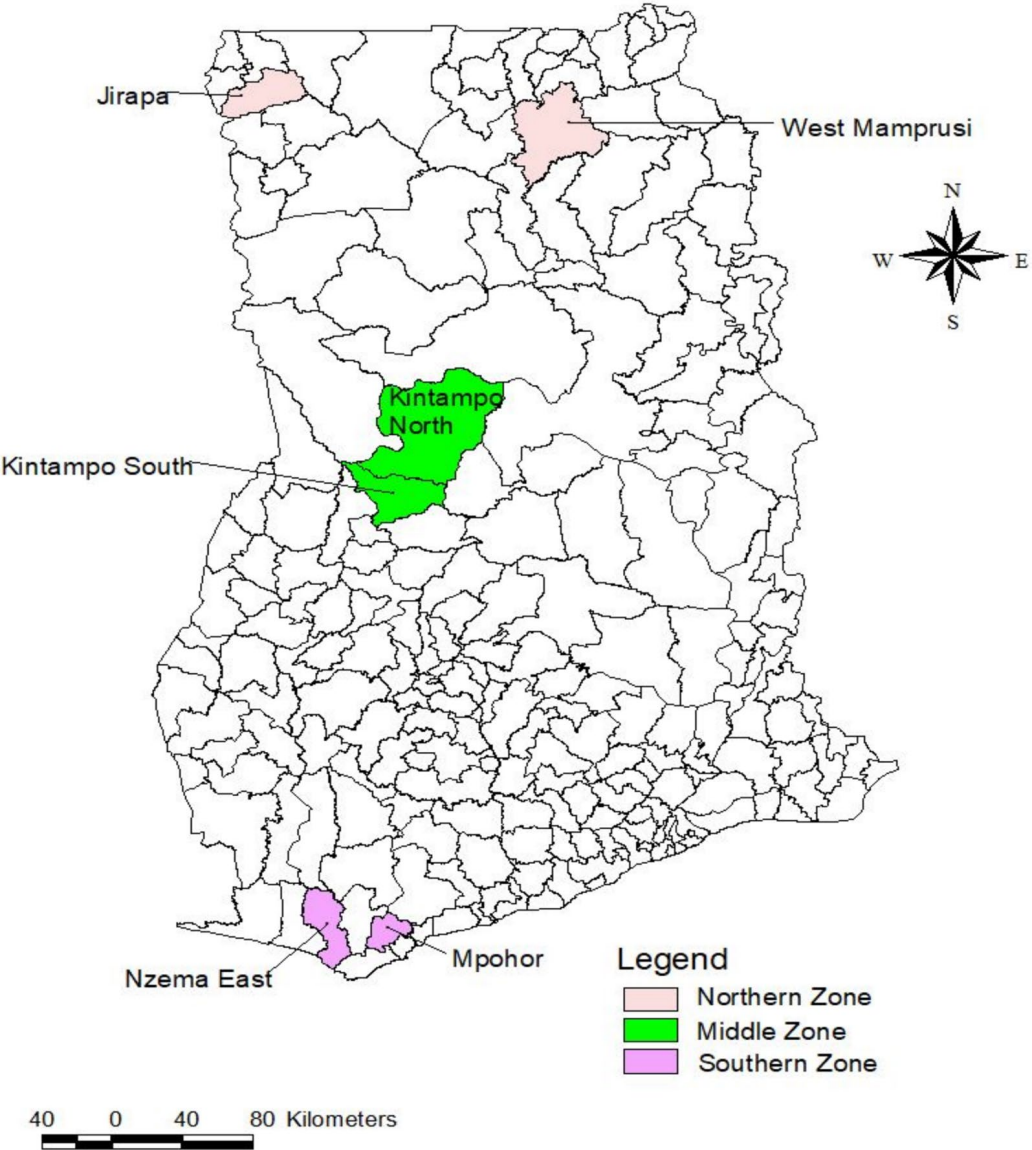
Map of Ghana showing the T3 study districts. Source: The Geographic Information Systems (GIS) unit of KHRC

Jirapa and West Mamprusi Municipalities are located in the north-western part of the Upper West Region and North East Regions, respectively. The vegetation of the two municipalities is Guinea Savanna woodland with light under growth and scattered trees. The major economic trees are Shea, Dawadawa, and Baobab species. The area has a combined population of 267,034 [10]. Malaria ranks highest as a major health problem. Public health facilities in the two municipalities include 2 municipal hospitals, 1 polyclinic, 11 health centers/clinics, and 35 CHPS compounds (Fig. 1).

### Sample size and sampling method

Two districts were conveniently selected from each of the 3 zones in the country. In each district, the district hospital, one health centre and 3 CHPS zones were selected, making it 5 health facilities per zone and a total of 30 health facilities across the six districts/municipalities from the 3 zones [12]. In each of the 30 health facilities, 20 client exit interviews were conveniently performed from each facility, as and when they exit the facility after been diagnosed and treated for malaria, making a total of 600 clients exit interviews taking into consideration 5% non-response rate.

### Data collection and management

18 data collectors and 3 supervisors were trained by senior research officers on the objectives of the study and the data collection methods. Data was collected using Redcap application. The data collection period spanned over 2 weeks from 11th November to 29th November 2019. The inclusion criteria for clients exit interview were for clients that have been diagnosed and treated for malaria. Data collectors were located at the facility exit point to enquire about this and interview participants who pass the inclusion criteria to be included and interviewed. The purpose of the study, the benefits and right of the participants, the procedure involved were explained to all participants of the study and were assured of confidentiality. In addition, voluntary informed consent was obtained from all participants by signing a consent form.

### Data analysis

Data were analyzed using Stata 17.0. To assess adherence to the T3 policy from the clients’ perspective, the study assessed; “Test” by asking whether the client was tested using malaria rapid diagnostic test RDT or microscopy, “Treat” by asking whether the client was prescribed a recommended antimalarial drug and “Track” by asking whether the client whether there was an indication of follow up assessment or any plan for a review. A facility was classified as adherent to the T3 policy if a client responded “Yes” to all the three questions. To assess adherence to the T3 policy at the facility level; “Test” was assessed by asking the facility in-charge whether they performed test for reported feverish conditions, “Treat” was assessed by asking whether the facility prescribed the required antimalarial for confirmed cases and “Track” was assessed by asking whether prescribers indicated any follow up measures or review measure to confirm surveillance after treatment.

Tables were used to describe participant background characteristics and chi square test to assess the association between background characteristics and adherence to the T3 policy. Unadjusted and adjusted odds ratios with 95% confidence interval using logistic regression models were performed to assess the factors associated with the T3 policy.

## Results

Overall, 590 patients with febrile illness and 30 facility managers were interviewed from 30 facilities in 6 districts across 3 epidemiological zones in Ghana. CHPS compounds formed the majority of the facilities surveyed. Twenty-nine out of 30 health facilities had RDT test kits and antimalarials (Table 2). In all, 30% of facility managers in the North and Middle zones indicated that their staff had not been trained adequately on the T3 policy compared to the Southern zone where all staff were reported to be trained. Table two, showed that all the facilities indicated having processes to track patients that have visited the facility for malaria diagnose and treatment.

**Table 2:** Background characteristics of health facilities surveyed across the three epidemiological zones in Ghana

Indicator	North n (%)	Middle n (%)	South n (%)	Total N (%)
	n = 10	n = 10	n = 10	N = 30
Facility type				
CHPS	6 (60.0)	6 (60.0)	6 (60.0)	18 (60.0)
Health Centre	1 (10.0)	2 (20.0)	3 (30.0)	6 (20.0)
Hospital	3 (30.0)	2 (20.0)	1 (10.0)	6 (20.0)
Test				
Microscopy services				
Yes	3 (30.0)	3 (30.0)	2 (20.0)	8 (26.7)
No	7 (70.0)	7 (70.0)	8 (80.0)	22 (73.3)
Test kits (malaria RDT)				
Yes	9 (90.0)	10 (100.0)	10 (100.0)	29 (96.7)
No	1 (10.0)	0 (0.0)	0 (0.0)	1 (3.3)
Treat				
Availability of antimalarial				
Yes	9 (90.0)	10 (100.0)	10 (100.0)	29 (96.7)
No	1 (10.0)	0 (0.0)	0 (0.0)	1 (3.3)
Training of personnel on T3				
Yes	7 (70.0)	6 (60.0)	10 (100.0)	23 (76.7)
No	3 (30.0)	3 (30.0)	0 (0.0)	06 (20.0)
Not known	0 (0.0)	1 (10.0)	0 (0.0)	1 (3.3)
Track				
Review after treatment				
Yes	10 (100.0)	10 (100.0)	10 (100.0)	30 (100.0)
No	0 (0.0)	0 (0.0)	0 (0.0)	0 (0.0)

**Fig. 2:**
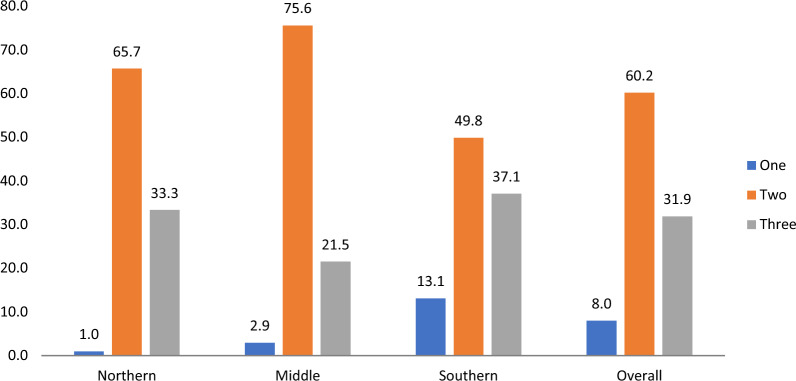
Percentage distribution of patients that adhered to one, two, or all three of the test, treat and track malaria policy

Figure 2 shows the number of patients and the actual T3 process, one (Test), two (Treat), and three (Track) are the T3s that were performed at the facilities in each zone. Of the number of participants observed in the northern zone 0.95% went through one component of the policy, 65.7% went through two processes and 33.3% went through the entire process. Distribution of the T3s in the middle zone is presented on the chart as; completion of only one component of the policy (5/2.9%), two components (130/75.6%), and three components (37/21.5%). Also in the Southern zone, T3s are given as one component (41/13.1%), two components (156/49.8%), and three components (114/37.1%). Overall, high (355/60.2%) indicated number of persons taken through any of the two processes, three processes (188/31.9%) and only one process (47/8.0%).

From Table 3, data on client interview review revealed significant variations across several variables. In terms of zones, adherence rates were highest in the Middle zone, with 78.5% of participants adhering, while the Northern zone showed a lower adherence rate of 66.7%. Ownership type also played a role; government facilities had a higher non-adherence rate (88.8%) compared to CHAG facilities where 41.5% adhered. Regarding sex, male participants had a slightly higher non-adherence rate (72.7%) than females (65.8%). Age groups showed mixed results, with the 10–19 age group having a non-adherence rate of 74.1%. Lastly, the type of health staff involved influenced adherence, particularly with physician assistants achieving a 71–96% adherence rate. Overall, these findings highlight the complex factors influencing client adherence across different demographics and healthcare settings.

**Table 3:** Association between background characteristics and adherence to the T3 policy from clients exit interviews at health facilities

Variables	n (%)	Client’s adherence		Chi-square	p-values
		Not adhered	Adhered		
Zones					
Middle	172 (29.2)	135 (78.5)	37 (21.5)		< 0.001
Northern	105 (17.8)	70 (66.7)	35 (33.3)	12.488	
Southern	313 (53.1)	197 (62.9)	116 (37.1)		
Ownership					
Government	524 (88.8)	364 (69.5)	160 (30.5)	5.367	0.068
CHAG	66 (11.2)	38 (58.5)	27 (41.5)		
Sex					
Male	198 (33.6)	144 (72.7)	54 (27.3)	2.894	0.089
Female	392 (66.4)	258 (65.8)	134 (34.2)		
Age					
10–29	415 (70.3)	288 (69.4)	127 (30.6)		0.364
30–44	115 (19.5)	72 (62.6)	43 (37.4)	2.019	
45+	60 (10.2)	42 (70.0)	18 (30.0)		
Facility type					
CHPS	347 (58.8)	224 (64.6)	123 (35.4)		0.079
Health centre	135 (22.9)	100 (74.1)	35 (25.9)	5.075	
Hospital	108 (18.3)	78 (72.2)	30 (27.8)		
Health staff/prescriber					
Community health nurse	364 (68.4)	226 (62.09)	138 (37.91)		0.069
Physician assistant	107 (20.1)	77 (71.96)	30 (28.04)	7.107	
Midwife	53 (1.0)	39 (73.6)	14 (26.4)		
Other	8 (1.5)	7 (87.5)	1 (12.5)		

**Fig. 3:**
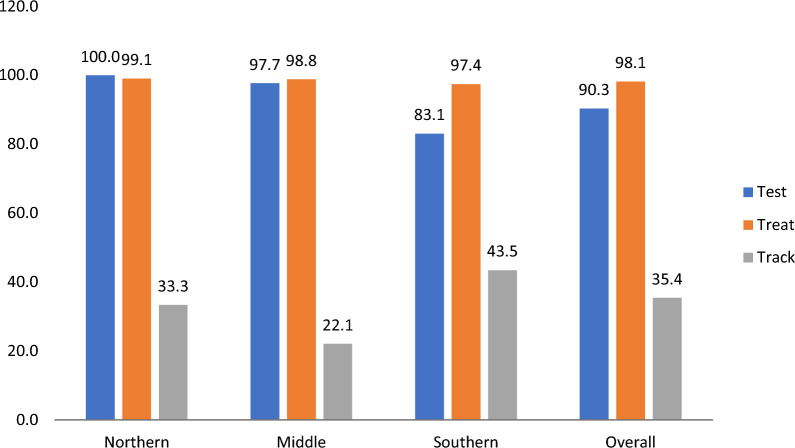
Proportions of patients with febrile illness who reported been tested, treated and informed to return for review (tracked) by epidemiological zones

Figure 3 describes the proportions of patients with febrile illness who reported been tested, treated and informed to return for review (tracked) by epidemiological zones. Over all 90%, 98% and 35% of patients indicated being tested, treated and tracked respectively. The track to the T3 policy has the lowest percentages ranging from 22 to 44% in the respective zones.

Table 4 describes the factors associated with adherence to the T3 policy from clients’ perspective.

**Table 4:** Factors associated with adherence to the T3 policy; clients’ perspective

Variables	Crude OR (95% CI)	p-value	Adjusted OR (95% CI)	p-value
Cluster/zone				
Middle	1		1	< 0.001*
North	1.82 (1.1, 3.1)	< 0.001*	1.74 (0.9, 3.3)	
South	2.15 (1.4, 3.3)		2.87 (1.7, 4.8)	
Facility type				
CHAG	1		1	0.057
Government	0.60 (0.4, 1.0)	0.526	0.5 (0.3, 1.0)	
Sex				
Female	1			0.018*
Male	0.72 (0.4, 1.1)	0.090	0.6 (0.4, 0.9)	
Age				
15–29	1			0.045*
30–44	1.35 (0.9, 2.1)	0.366	1.9 (1.1, 3.0)	
45+	0.97 (0.5, 1.8)		1.1 (0.6, 2.1)	
Classification of facility				
CHPS compound	1			0.030*
Health centre	0.64 (0.4, 1.0)	0.080	0.5 (0.3, 0.8)	
Hospital	0.70 (0.4, 1.1)		0.8 (0.4, 1.5)	
Health staff				
Community health nurse	1			0.088
Medical physician assistant	0.64 (0.4, 1.0)	0.078	0.9 (0.5, 1.8)	
Midwife	0.59 (0.3, 1.1)		0.5 (0.2, 0.9)	
Other	0.23 (0.0, 2.0)		0.2 (0.0, 1.9)	

^*^ Significant at 0.05

The analysis of factors associated with adherence to the T3 Policy from the clients’ perspective indicates several significant variables. In terms of geographic zones, individuals from the North and South showed increased odds of adherence compared to the Middle zone, with adjusted odds ratios (OR) of 1.74 and 2.87, respectively, highlighting a strong association (p < 0.001 for South). Facility type revealed that government facilities had lower odds of adherence (adjusted OR 0.5, p = 0.057) compared to CHAG facilities. Gender differences were notable, with females having higher odds of adherence (adjusted OR 1) and males exhibiting lower odds (adjusted OR 0.6, p = 0.018). Age also played a role; individuals aged 30–44 had an increased likelihood of adherence (adjusted OR 1.9, p = 0.045), while those aged 45 and older did not show significant odds. Regarding the classification of facilities, CHPS compounds indicated a baseline adherence, with health centres and hospitals showing lower adjusted odds (0.5 and 0.8, respectively, p = 0.030). Lastly, among health staff categories, midwives had an adjusted OR of 0.5 (p = 0.078), suggesting potential influence on adherence. Overall, these findings underscore the complexity of adherence factors, emphasizing the significance of zone, sex, age, and facility type.

## Discussion

Ghana National Malaria Elimination Programme (NMEP) in line with the WHO aims to minimize malaria morbidity. It aims to approve access to vector control, diagnosis, and treatment, as well as strengthen surveillance and reporting [13]. This study assessed the adherence to the T3 policy initiated by the WHO for effective malaria diagnoses and management. It was done through facility assessment and clients’ perspective on the policy.

### Testing

The results showed that each of the facilities in almost all the three zones have access to RDT or microscopy for testing cases, with about 96.7% rate at the facility level, while results from the clients exit interview showed over all 90%of confirmed testing before treatment. However, the lowest rate of testing among the three zones from client’s perspective was the southern zone which recorded 83.1%. The results from this study are higher as compared to a study in Uganda which showed that 86% of the health facilities were testing over 75% of patients suspected of malaria [14]. The increase in testing rate among health facilities may due to increase campaigns raising awareness about malaria symptoms, leading more people to seek testing, expansion of healthcare facilities and testing is often integrated into routine health services, making it more accessible.

Some studies have shown that public primary healthcare facilities lack diagnose kits such as microscopes, and inadequate personnel to use the kits [15]. Frequent stock out of logistics such as RDT kits and reagent for microscopy and the absence of qualified staff with malaria testing capacity hinder effective compliance with the components of the T3 policy. A study conducted in Mozambique on diagnostic test kits supply chain revealed 17% of health centers experienced RDT stuck out in any given month. Hence, situation of nonexistence of microscopies, adherence to T3 policy remains a challenge [16]. The result of this study showed that more than 70% of the facilities across all zones have no access to microscopy test but have access to RDTs, which indicates that in terms of RDT stockout, most facilities may not be able to comply with the policy guidelines, in line with the finding of a study among patients in Zambia showing that continues availability of the testing tool has potential to improve adherence to test results and general malaria case management [17].

### Treatment

Data from the facility assessment showed that 97% of the facilities treated confirmed malaria episode with the required antimalaria, whiles the results from the clients’ interview revealed 98% of treatment with antimalarials, with the southern zone recording the least of 97.4%. The high treatment rate of the study results is similar to a qualitative study conducted in 16 health facilities in six districts in Ghana, where study participants expressed the relucted of prescribers to change from presumptive treatment to a new treatment guideline [18]. It was also evident from the study that 30% of the personnel from the north and middle zones indicated not been trained on the policy, which is high compared to a study in the Bongo district of Ghana which evaluated implementation challenges of the T3 policy. This could be due to high staff turnover which may have hindered adequate training for new staff, limited financing and resources which may impede training programs, resulting in fewer persons been taught, while inadequate healthcare infrastructure, particularly in remote areas, might limit access to training.

The T3 policy discourages presumptive treatment of malaria-based symptoms and signs alone. However, the study found out that apart from the northern zone, the other zones had clients treated for malaria without confirmatory testing. It has been reported that in Kenya, less than 40% of febrile children under 5 years were tested for malaria whiles in Ghana, 73% of children were presumptively diagnosed and treated for malaria [6]. In addition, fever from most endemic countries associated with malaria was diagnoses and treated presumptively as malaria [15]. Even though there is an association between fever malaria parasitaemia, it has been proven that not all patients presenting with fever have malaria parasites and that several other pathogens account for non-malaria fevers even in malaria endemic settings of Ghana [19]. It is, therefore, inappropriate to treat feverish conditions with ACT without confirming diagnoses of malaria with RDT or microscopy test [20]. Testing for malaria also provides an opportunity for improved diagnoses and better case management [15].

A study on health worker compliance with a “test and treat” malaria case management protocol conducted in Papua New Guinea revealed frequent prescriptions of antimalarial despite negative tests as the most reported barriers to policy compliance [21]. Compared with the results of the study showed that facilities in the middle and the southern zone provided antimalarials more than the required number of tests performed, meaning some prescribers from these facilities continue to provide ACT for treatment of malaria case without following the required guidelines, contrary to the enhancement of the policy practice. The outcome from the logistic regression analysis showed that some facilities in the south are prescribing malaria drug without testing, confirming a finding from qualitative research on malaria case management among prescribers in Ghana, which revealed that majority of the health facilities, especially the CHPS compounds lack laboratories capable of diagnosing malaria event, therefore, RDTs are mostly used at these levels. RDT kits can be hard to come by at health centers without labs and may affect the adherence level [18].

### Tracking

At the facility level, almost 100% responded with an indication of having processes of tracking for treatment outcomes by way of either home visit or facility review, whiles the clients exit interview found that only 35% of the patients were tracked according to T3 policy across all zones. This is higher compared to a study on compliance to the T3 policy in the Bosomtwi district in Ghana, which showed lack of malaria thresholds and monthly monitoring charts for cases and deaths, as well as restricted analysis on malaria data, which undermines the tracking component of the T3 implementation process [22]. Health facilities in the southern zone of Ghana had almost threefold increased odds of adhering to the policy relative to their counterparts in the middle and the northern zones. This may be due the fact that facilities in the south are mostly well equipped, have more qualified clinical and laboratory staff and located mainly in towns which may be closer to settlement areas, thereby making them easily accessible compared to those in the north and the middle zones where road network and distances to facilities remain a challenge. A study on guiding placement of health facilities in the Bunkpurugu-Yunyoo district in the northern Ghana, confirmed that for malaria case, location of the facility has a significant impact on malaria prevalence and care-seeking [23]. The results as well showed that CHPS compounds indicated a baseline adherence, with health centers and hospitals showing lower adjusted odds. This could be due to the fact that CHPS compounds are designed to be more accessible to rural communities, reducing travel time and cost for patients compared to health centers that may be located further away, emphasizes community involvement and volunteerism, which fosters trust and encourages local participation in health services and community health officers (CHOs) and volunteers are often from the same communities, which enhances the acceptance and utilization of services.

### Adherence

The findings indicate that the location of the health facility has influence on the entire process of adherence with the policy and males are likely to default on surveillance of treatment outcomes. Male counterpart may prioritize economic activities over health-related policies, and if men do not perceive malaria as a threat, they may not prioritize adherence to preventive measures. Overall, it was evident that the testing and treating components of the policy were high across all the zones, whiles tracking was low in all the zones for the clients exit interview. Results from the study revealed 32% of the patients adhered by completing the three processes of the policy. These findings are consistent with a study conducted in 2020 at the Mfantseman municipality in the central region of Ghana, which found 30%adherence level to the T3 policy among 414 febrile outpatients seen by 18 prescribers [24]. Clients’ low adherence rate could be attributed to lack of education about the significance of follow up, transportation challenges, or cultural attitudes. There is also the fear of stigma associated with malaria, ineffective communication between healthcare providers and patients can lead to misunderstandings about the importance of tracking. In rural areas, geographical barriers can limit access to healthcare facilities, making tracking difficult.

The level of adherence observed in this study was below that observed from a similar study by Agandaa et al*.* in the Bongo district of Ghana which revealed 42.5% adherence to the T3 policy among 353chilren from 28 health facilities. This could be from the fact that parents are more likely to send their children back for facility reviews because they are vulnerable, compered to adult males who may have refused facility review for unknown reasons. It was also evident that government health facilities had higher non-adherence rate (88.8) compared CHAG facilities, where 41.5% adhered to the policy. CHAG facilities often have better access to resources, including funding and medical supplies, which could enhance their testing capabilities. In addition, CHAG facilities may prioritize staff training and retention, leading to more knowledgeable and motivated healthcare providers, have strong community ties which could improve patient trust and encourage adherence to testing protocols. However, the 41.5% results of this study is higher with regards to a study on national treatment guidelines, which showed that only 20% of private drug outlet adhered to Kenya national malaria treatment guidelines [25].

Comparing the level of adherence of the T3 policy, a study in the Bosomtwi district of Ghana which evaluated compliance to T3 policy revealed 64%of malaria cases been tested before treatment using antimalarials [22]. The finding is relative lower as compared to what this study found, with evidence of 90% and 98% tested and treated cases respectively across the zones using ACT. Another study on adherence of the T3 policy on caregivers of children under 10 visiting the Over-the-Counter Medicine Sellers (OTCMS), revealed 56.6% adherence among OTCMS, and indicted that monitoring and supervision of OTCMS especially in the rural areas would lead to improving adherence of the policy and subsequently scaling up management of malaria events [26]. The results from that research were high compared to the adherence level revealed by this study.

### Limitations

There was potential limitation regarding recall bias during exit interview of clients, who may have forgotten about some of the information received at the health facility. The kind of treatment given may not have been accurately provided in the responses. This limitation was minimized by interviewing clients immediately they were exiting from health facilities. Also, healthcare managers as well as clients may have provided responses solely to please interviewers, which could lead to social desirability bias. However, we verified most of the responses from the facilities to reduce this risk through observations at the facility. Limited information on clients’ demographic characteristics like marital status, occupation, religion and wealth quintiles prevented further analysis on some potentially important independent variables.

## Conclusion

The study showed that testing and treating for malaria was high among health facilities across the three zones for both facility and clients’ assessment. However, from the clients’ perspective tracking of patients was very low across the three zones. Adherence was low especially for males who were more likely to default facility return for review or home monitoring. Training on the T3 policy was relatively inadequate especially for staff in the Northern and the Middle zones.

### Recommendations

In order to improve adherence to the T3 policy, the National Malaria Elimination Program (NMEP) should make sure that health facility employees receive sufficient periodic training, particularly those working in Ghana's Northern and Middle zones. It should also improve monitoring and supervision of health facilities. To help clients comprehend the value of monitoring, the NMEP might also step up client sensitization initiatives, particularly aimed at male clients.

## Data Availability

Information on data could be provided upon acceptable request.

## Abbreviations

WHO: World Health Organization
CHPS: Community Health and Planning Services
RDT: Malaria rapid diagnostic test
NMEP: National Malaria Elimination Programme
ACT: Artemisinin-based combination therapy
T3: Test, treat and track
OTCMS: Over the Counter Medicine Sellers
RDD: Research and Development Division
KHRC: Kintampo Health Research Centre
NHRC: Navrongo Health Research Centre
DHRC: Dodowa Health Research Centre
ARHR: Alliance for Reproductive Health Right
ADRRO: Anglican Diocesan Development Relief Organization
